# Transcriptomics of Environmental Enrichment Reveals a Role for Retinoic Acid Signaling in Addiction

**DOI:** 10.3389/fnmol.2016.00119

**Published:** 2016-11-16

**Authors:** Yafang Zhang, Fanping Kong, Elizabeth J. Crofton, Steven N. Dragosljvich, Mala Sinha, Dingge Li, Xiuzhen Fan, Shyny Koshy, Jonathan D. Hommel, Heidi M. Spratt, Bruce A. Luxon, Thomas A. Green

**Affiliations:** ^1^Center for Addiction Research, The University of Texas Medical Branch, GalvestonTX, USA; ^2^Department of Pharmacology and Toxicology, The University of Texas Medical Branch, GalvestonTX, USA; ^3^Mitchell Center for Neurodegenerative Diseases, The University of Texas Medical Branch, GalvestonTX, USA; ^4^Department of Biochemistry and Molecular Biology, The University of Texas Medical Branch, GalvestonTX, USA; ^5^Biomedical Informatics Program, The University of Texas Medical Branch, GalvestonTX, USA; ^6^Sealy Center for Molecular Medicine, Institute for Translational Science, The University of Texas Medical Branch, GalvestonTX, USA; ^7^Department of Preventive Medicine and Community Health, The University of Texas Medical Branch, GalvestonTX, USA

**Keywords:** drug dependence, stimulants, RNA-seq, differential rearing, self-administration, regionally enhanced gene expression

## Abstract

There exists much variability in susceptibility/resilience to addiction in humans. The environmental enrichment paradigm is a rat model of resilience to addiction-like behavior, and understanding the molecular mechanisms underlying this protective phenotype may lead to novel targets for pharmacotherapeutics to treat cocaine addiction. We investigated the differential regulation of transcript levels using RNA sequencing of the rat nucleus accumbens after environmental enrichment/isolation and cocaine/saline self-administration. Ingenuity Pathways Analysis and Gene Set Enrichment Analysis of 14,309 transcripts demonstrated that many biofunctions and pathways were differentially regulated. New functional pathways were also identified for cocaine modulation (e.g., Rho GTPase signaling) and environmental enrichment (e.g., signaling of EIF2, mTOR, ephrin). However, one novel pathway stood out above the others, the retinoic acid (RA) signaling pathway. The RA signaling pathway was identified as one likely mediator of the protective enrichment addiction phenotype, an interesting result given that nine RA signaling-related genes are expressed selectively and at high levels in the nucleus accumbens shell (NAcSh). Subsequent knockdown of *Cyp26b1* (an RA degradation enzyme) in the NAcSh of rats confirmed this role by increasing cocaine self-administration as well as cocaine seeking. These results provide a comprehensive account of enrichment effects on the transcriptome and identify RA signaling as a contributing factor for cocaine addiction.

## Introduction

Some people experimenting with cocaine become addicted at first exposure, while others are resistant, even after many exposures. These important individual differences in vulnerability to addiction are a function of the interaction between genes and the environment (McGue et al., 1996). Genetic factors that play an essential role in individual differences in susceptibility to drugs of abuse are well studied; however, environmental influences on gene expression is an area in need of further study (Thiriet et al., 2008).

Environmental enrichment is a non-drug, non-surgical, non-genetic manipulation producing a protective addiction phenotype in rodent models (Bardo et al., 2001; Green et al., 2002, 2010; Chauvet et al., 2009; Thiel et al., 2009, 2011; Solinas et al., 2010; Alvers et al., 2012; Chauvet et al., 2012; Nader et al., 2014). In the enriched condition (EC), animals are group-housed with access to children’s plastic toys which are changed and rearranged daily, while those in the isolated condition (IC) are single-housed without disturbance. In drug self-administration studies, EC rats self-administer less cocaine than IC rats in the acquisition, maintenance, extinction, and reinstatement phases of cocaine self-administration (Green et al., 2010). Understanding the mechanisms underlying the protective phenotype of environmental enrichment may help uncover novel pharmacotherapeutic targets for prevention and treatment of addiction.

The nucleus accumbens (NAc) is an essential brain region for reward (among several regions) and altering the expression of genes in the NAc shell affects cocaine self-administration in rats (Green et al., 2010; Larson et al., 2011; Zhang et al., 2014). First we utilize quantitative RNA sequencing to analyze expression of 14,309 transcripts in the NAc of EC and IC rats self-administering cocaine or saline. Next, a transcriptomic analysis of topographical gene expression was performed to identify genes expressed selectively in the mouse NAc shell, using tools and data from the Allen Brain Atlas^1^. The convergence of RNA-seq and topographical gene expression analyses pointed clearly to retinoic acid (RA) signaling as the most promising pathway to target.

As the active metabolite of vitamin A, RA acts as an essential molecule in multiple biological processes, such as embryonic development (Rhinn and Dolle, 2012), immune response (Mielke et al., 2013), cell proliferation and differentiation (Chen et al., 2014), and maintenance of the nervous system (Maden, 2007). There is increasing evidence that RA plays an important role in the adult brain (Maden, 2007). Although no study has yet reported on the role of RA in addiction-related behavior *per se*, there is some evidence that double null mutants of the RA receptor β (RARβ) with the retinoid X receptor RXRβ or RXRγ decrease dopamine D2 receptor expression selectively in the shell of the NAc (Krezel et al., 1998). These rats displayed decreased cocaine-stimulated locomotor activity; however, they also had severe decrements in the rotarod task and spontaneous locomotor activity.

Retinoic acid is highly concentrated in the brain, including the striatum (Kane and Napoli, 2010). RA is synthesized in the cytoplasm in two steps: first, retinol is oxidized to retinaldehyde via retinol dehydrogenase (*Radh a.k.a. Adh*). Then, retinaldehyde is irreversibly converted to RA through retinaldehyde dehydrogenase (*Raldh* a.k.a. *Aldh1a1-3*). Excess RA is degraded by *Cyp26b1* into polar metabolites (Maden, 2007; Chen et al., 2014). We show here that knockdown of *Cyp26b1* via a novel adeno-associated viral vector increases cocaine self-administration.

## Materials and Methods

### Animals

For the RNA-seq study, male Sprague-Dawley rats (Harlan, Houston, TX, USA) arrived at 21 days of age and were enriched or isolated for 30 days before behavioral testing. EC rats (*n* = 20) were group-housed in a large metal cage (70 cm × cm 70 × 70 cm) with 14 hard children’s plastic toys changed and rearranged daily. This density (245 cm^2^/rat) was higher than a majority of environmental enrichment studies, but was still more than double that required by NIH. EC rats were split into two cages after 50 days of age. IC rats (*n* = 20) were single housed in standard polycarbonate cages. These conditions produce a resistant (i.e., EC) and susceptible (i.e., IC) behavioral addiction phenotype (Green et al., 2002, 2003, 2010). While it is true that short term isolation is a stressor, enriched rats (even at lower density) show greater signs of chronic stress, even though these rats do not show outward signs of stress (Crofton et al., 2015). Rats remained in these homecage conditions throughout all behavioral tests, except during testing. For vector injection and behavioral tests, male Sprague-Dawley rats were obtained at 225–249 g. Rats were pair-housed and maintained in a controlled environment (temperature, 22°C; relative humidity, 50%; and 12 h light/dark cycle, lights on 0600 h) in an Association for Assessment and Accreditation of Laboratory Animal Care (AAALAC) approved colony and procedures were approved by the UTMB Institutional Animal Care and Use Committee and conform to the NIH Guide for the Care and Use of Laboratory Animals.

### Intravenous Cocaine Self-Administration with Environmental Enrichment

Rats were anesthetized with ketamine (100 mg/kg, IP) and xylazine (10 mg/kg, IP), and implanted with an indwelling Silastic catheter (0.2 mm I.D.; Fisher Scientific, Pittsburgh, PA, USA) into the jugular vein. The catheter passed under the skin to exit on the rat’s back. The catheters were infused with 0.1 ml of a sterile saline solution containing heparin (30.0 U/ml), ticarcillin (250,000 U/ml) and streptokinase (8000 IU/ml) daily, to prevent infection and maintain catheter patency throughout the duration of experiments.

**FIGURE 1:**
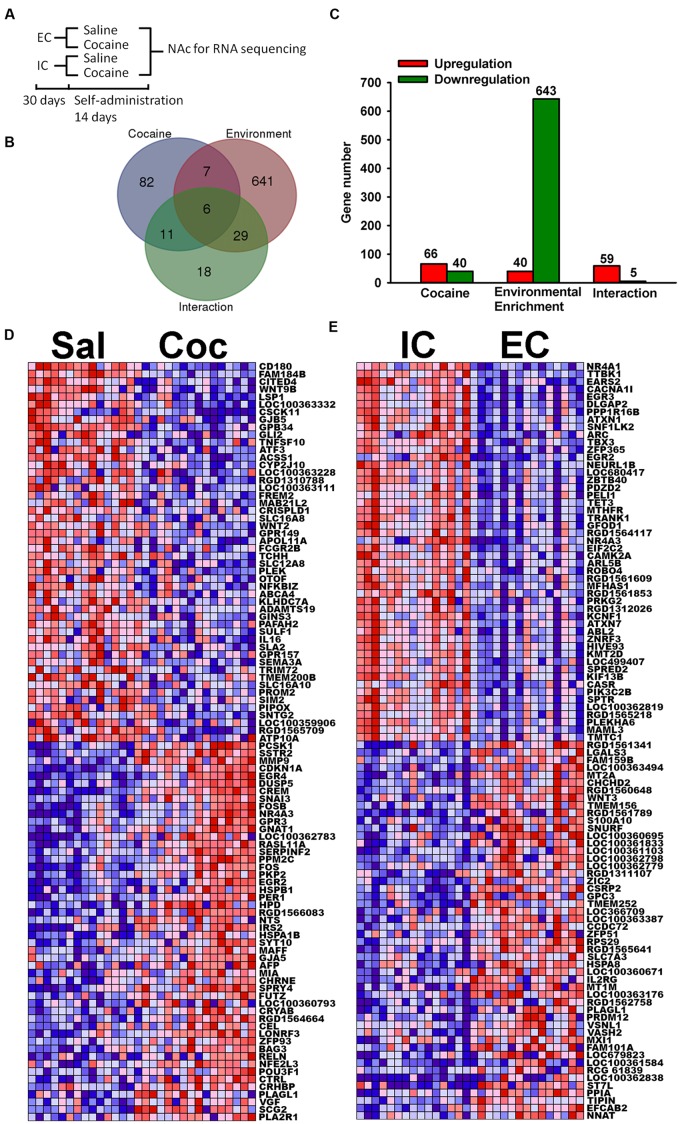
**Most highly regulated transcripts.** **(A)** Schematic diagram of RNA sequencing experiment. **(B)** Venn diagram of transcripts that are significantly regulated at the *p* < 0.001 level by Cocaine, Environmental Enrichment, and their interaction. **(C)** Bar chart showing the number of transcripts that are up or down regulated. **(D)** Heat maps of top 50 upregulated and downregulated transcripts by cocaine. Red represents high expression and blue represents low expression. **(E)** Heat map of top 50 upregulated and downregulated transcripts by environmental enrichment. IC stands for isolated condition. EC stands for enriched condition.

One week after catheter surgery, rats were allowed to self-administer 0.5 mg/kg/infusion cocaine (National Institute on Drug Abuse, Bethesda, MD, USA) or saline under a fixed ratio 1 (FR1) schedule for 2 h/day for 14 days. The session terminated when the rat received 30 infusions to eliminate cocaine intake differences between EC and IC rats. Tissue was harvested 3 h after the beginning of the last session (**Figure 1A**).

### Quantitative RNA Sequencing

Rat brains were harvested 3 h after the beginning of the last self-administration session and the left side of the NAc was dissected on an ice-cold platform for mRNA analysis. The right side was used for protein quantification (Lichti et al., 2014). The RNA was extracted and purified with the RNeasy mini kit (Qiagen, Valencia, CA, USA). cDNA libraries were created by reverse transcribing the RNA and creating the second strand. Blunt ends were phosphorylated and “a-tailed” so that adapters could be ligated to both ends. Adapters were individually “bar coded” and thus samples were not pooled despite having 4 samples per flow cell, yielding *n* = 7–8 for each condition (total *N* = 30). RNA was sequenced with a HiSeq 1000 system from Illumina. cDNA was amplified using “bridge” amplification. Base calls were made using fluorescently labeled nucleotides. More than 100 million reads with 50 bp (paired-end reads) were mapped for each rat and quality was checked with FastQC (v0.9.1) (Andrew, 2010). Reads were mapped to the rat reference genome (RN4) using Tophat2 (v2.0.4) (Kim et al., 2013) and Bowtie2 (v2.0.0.6) (Langmead and Salzberg, 2012) software packages. The R package EdgeR (v.3.0.8) (Anders and Huber, 2010; Robinson et al., 2010) was then used for analysis using the log-transformed “trimmed mean for *M*-values” (TMM) method for normalization and tag-wise dispersion using “count” data. A likelihood ratio *F*-test was used for generating *p*-values to compare EC vs. IC rats and cocaine vs. saline. Cross-validation of RNA-seq results was achieved by looking at correlation of expression between qPCR and RNA-seq for Fabp5 (Forward: 5′ CTTGCACCTTGGGAGAGAAG 3′, Reverse: 5′ CATCTTCCCGTCCTTCAGTT 3′) and Hspa5 (Forward: 5′ AACCAAGGATGCTGGCACTA 3′; Reverse: 5′ ATGACCCGCTGATCAAAGTC 3′), with the qPCR being normalized to Reep5 (Forward: 5′ GGTTCCTGCACGAGAAGAACT 3′; Reverse: 5′ GAGAGAGGCTCCATAACCGAA 3′) (**Supplementary Figure S6**). The Fabp5 was chosen because it is in the RA pathway and selectively expressed in the NAc shell, both aspects central to this paper. Hspa5 (Bip) was chosen because it has extremely high expression levels, and because we have studied this transcript with cocaine in the past (Pavlovsky et al., 2013). Using qPCR to cross-validate the RNA-seq data is somewhat problematic. Our conclusion is that using a less accurate and less precise method to validate a more accurate and precise method is difficult at best. The problem encountered is that qPCR needs to be normalized to a control or “housekeeping” transcript. The variance from the transcript of interest gets compounded with the variance inherent in the housekeeping gene, increasing variance. Multiple transcripts were assessed for normalization and the end results were quite different depending solely upon which normalization gene was chosen. Many traditional “housekeeping” transcripts were regulated (e.g., *gapdh*) or trended toward regulation (e.g., β-actin) by enrichment and/or cocaine. In the end, Reep5 was chosen because it was a housekeeping gene not regulated by cocaine or enrichment. However, the variability of the Reep5 expression among samples washed out main effects.

The quantitative transcriptomic data have been deposited in NCBI’s Gene Expression Omnibus (Edgar et al., 2002) and are accessible through GEO Series accession number GSE88736^2^.

### Ingenuity Pathway Analysis (IPA)

In order to study the biological functions and pathways regulated by cocaine and environmental enrichment, transcripts significantly regulated (*p* < 0.05) were analyzed with IPA. Canonical Pathways, Upstream Regulators, Diseases and Biological Functions, and Networks were used to identify significantly regulated transcript sets. Because regulation of any given gene could be a statistical anomaly (i.e., false positive), bioinformatic analyses have been developed under the assumption that regulation important for function will occur in a coordinated fashion at multiple targets within a given pathway. Thus, the IPA analysis assesses over-representation of multiple targets within known pathways. For this analysis, the list of significantly regulated genes is further analyzed for significantly regulated pathways, thus minimizing the effect of individual false positives. Preliminary analysis of ribosomal proteins, tyrosine phosphatases, de-ubiquitinating enzymes and proteasomal proteins, all of which show highly coordinated regulation (see **Supplementary Figures S4A,B**) demonstrate that the *p* < 0.05 cutoff does **not** produce an overabundance of false positives.

### Gene Set Enrichment Analyses (GSEA)

To complement the IPA analysis and avoid the problems of Type I vs. II error, normalized expression intensity (normalized by EdgeR using TMM) of all identified transcripts were analyzed by GSEA. The normalized enrichment score (NES) indicates the degree to which this gene set is overrepresented at the top or bottom of the ranked list of genes in the expression dataset (Subramanian et al., 2005). In IPA analyses, a p-value cutoff is required to decide whether transcription is significantly regulated or not. The problem of setting a cutoff is that high stringency introduces false negative results (i.e., high Type II error), whereas low stringent cutoffs introduce false positive results (i.e., high Type I error). To complement the IPA analysis and avoid the problem of Type I vs. II error, normalized expression intensity of all identified transcripts were analyzed by GSEA. In the GSEA analysis, all transcripts are ranked by a signal2noise metric (the difference of means scaled by the standard deviation), and the significance of a given set of genes is determined using a running-sum statistic to determine rank-order over-representation. Thus, this analysis produces a statistic at the gene set level without the need for a p-value cutoff. Gene sets were from the Broad Institute‘s set (v.4.0) of curated gene sets [C2.all], gene ontology gene sets [C5.all] and transcription factor target gene sets [C3.tft]. In GSEA, the Enrichment Score (ES) for the gene set is the degree to which this gene set is overrepresented (i.e., “enriched”) at the top or bottom of the ranked list of genes in the expression dataset. NES indicates the enrichment score for the gene set after it has been normalized to adjust for size of the gene set.

### Quantitative Proteomics

A secondary analysis of our previously published liquid chromatography tandem mass spectrometry (LC-MS/MS) protein data from these same rats (Lichti et al., 2014) was used to corroborate RNA results where appropriate.

### Topographic Transcriptomic Analysis

The regional specificity of gene expression within the brain strongly suggests that genes are not expressed where they are not needed. Thus, *in situ* visualization of regional expression patterns in brain slices can provide a topographical map of enhanced gene expression for each specific brain region. One could then hypothesize that genes with regionally enhanced expression patterns in the NAc shell would likely be important for addiction-related behavior. Accordingly, the Allen Brain Institute’s Anatomic Gene Expression Atlas Gene Finder^3^ (Lein et al., 2007) algorithm was used with coronal image sets, seed coordinates of 4200, 5400, 6400 (right side) and 4200, 5600, 5000 (left side) with an expression threshold of 1. This Atlas defines mouse brain expression patterns; however, there is good concordance in expression patterns among mice, rats and humans (Zetterstrom et al., 1999; Strand et al., 2007). The genes with a regional enrichment score of 1.25 fold or higher (>250 genes) were then culled by individual manual inspection to remove genes whose enrichment fold change was clearly driven by experimental artifact such as bubbles, debris, misalignment and high expression in nearby regions (e.g., Islands of Calleja, NAc core, olfactory tubercles, etc.) to yield 178 genes. These 178 genes were submitted to IPA analysis. Once the RA signaling pathway was identified, manual curation of sagittal sets for RA signaling genes without coronal sets was used to identify additional targets (Rbp1 and Rdh10) with shell-specific expression. Images for **Figures 6A–D** were taken from the Allen Brain Atlas:

**STRA6**: http://mouse.brain-map.org/experiment/siv?id=75041492&imageId=74948838&initImage=ish&coordSystem=pixel&x=5288.5&y=3704.5&z=1

**FABP5**: http://mouse.brain-map.org/experiment/siv?id=70634396&imageId=70561807&initImage=ish&coordSystem=pixel&x=4104.5&y=3104.5&z=0

**RBP1**: http://mouse.brain-map.org/experiment/siv?id=68076899&imageId=68005433&initImage=ish&coordSyste&imgeId=68005433&initImage=ish&coordSystem=pixel&x=7448.5&y=4096.5&z=1

**RDH10**: http://mouse.brain-map.org/experiment/siv?id=75831736&imageId=75791261&initImage=ish&coordSystem=pixel&x=7472.5&y=4040.5&z=1

**ALDH1A3:** http://mouse.brain-map.org/experiment/siv?id=75861799&imageId=75806893&initImage=ish&coordSystem=pixel&x=6872.5&y=3496.5&z=1

**CYP26B1:** http://mouse.brain-map.org/experiment/siv?id=72081548&imageId=71940737&initImage=ish&coordSystem=pixel&x=4280.5&y=3160.5&z=1

**RARβ:** http://mouse.brain-map.org/experiment/siv?id=75038442&imageId=74930074&initImage=expression&colormap=0.5,1,0,256,4&coordSystem=pixel&x=4416.5&y=2952.5&z=1

Finally, a literature search of published papers with *in situ* and immunohistochemical images was used to confirm Allen Brain Atlas expression and identify additional shell-specific expression components of the RA signaling pathway (McCaffery and Drager, 1994; Zetterstrom et al., 1999).

### Adeno-Associated Virus knockdown of *Cyp26b1*

In order to knock down CYP26b1 expression, five 24-nucleotide sequences were identified within the CYP26b1 mRNA sequence (Ensembl transcript ID: ENSRNOT00000020505) using the criteria previously described (Hommel et al., 2003; Benzon et al., 2014) (**Supplementary Table S1**). The oligonucleotide sequences were synthesized and the annealed hairpin oligonucleotides were cloned into pAAV-shRNA plasmids (pAAV-*Cyp26b1*shRNA). Hairpin expression from these plasmids was driven by the mouse U6 promoter using a pol-III mechanism. In addition to the hairpin, enhanced green fluorescent protein (eGFP) was expressed from a separate expression cassette driven by a pol-II promoter (CMV).

In order to determine the most effective hairpin, all five hairpins were screened *in vitro*. Since HEK293 does not express rat *Cyp26b1*, a *Cyp26b1* overexpression plasmid was constructed. To create the *Cyp26b1* overexpression plasmid to test knockdown efficiency, the rat CYP26b1 gene sequence was amplified from rat genome cDNA using polymerase chain reaction (forward primer: TAGGAATTCCTCCTGGGTTTCTTCGAGGG; Reverse: TAGGTCGACATCCAAGAGGGTGGGAGTCA) and cloned into the pAAV-IRES-hrGFP plasmid (Agilent Technologies, Santa Clara, CA, USA). The various pAAV-*Cyp26b1****-s***hRNA plasmids or pAAV-Control shRNA plasmid was co-transfected with pAAV-*Cyp26b1*-IRES-hrGFP plasmid into HEK-293 cells using FuGENE^®^ 6 Transfection Reagent (Promega)/Lipofectamine 2000 (Life Technologies, Grand Island, NY, USA).

The cells were harvested 24–48 h later, followed by RNA extraction and reverse transcription to cDNA. The RNA was extracted using RNeasy Mini Kit (Cat No. 74104). Contaminating DNA was removed (TURBO DNA- Free, Life Technologies, Carlsbad, CA, USA) and 5 μg total RNA was reverse transcribed into cDNA (SuperScript III First Strand Synthesis: Invitrogen catalog # 18080051). Relative knockdown was measured with Real-time PCR (SYBR Green: Applied Biosystems, Foster City, CA, USA) on an Applied Biosystems 7500 fast thermocycler with *Cyp26b1* qPCR primers (forward: CCAGCAGTTTGTGGAGAATG; Reverse: GTCCAGGGCGTCTGAGTAGT). The results were normalized to *Gapdh* (forward: AACGACCCCTTCATTGAC; reverse: TCCACGACATACTCAGCAC). All primers were validated and analyzed for specificity and linearity prior to experiments (Alibhai et al., 2007).

Efficiency of hairpin was further validated by western blot. HEK293 cells were homogenized in a buffer containing sucrose, Hepes buffer, sodium fluoride, 10% SDS, and protease and phosphatase inhibitors (Sigma–Aldrich: P-8340, P-2850, P-5726). Protein concentration was assessed using the Pierce BCA Protein Assay Kit (Thermo Fisher Scientific, Waltham, MA, USA). Protein samples were denatured at 95° for 10 min and run on a 12% gel (Criterion TGX, Bio-Rad Laboratories, Hercules, CA, USA) then transferred to a polyvinylidene fluoride (PVDF) membrane (Millipore, Billerica, MA, USA). The membrane was blocked with blotting-grade blocker (non-fat dry milk), incubated with Cyp26b1 primary antibody (rabbit, 1:500, 21555-1-AP, Proteintech, Rosemont, IL, USA) and GAPDH primary antibody (mouse, 1:10000, Abcam, Cambridge, MA, USA), washed with TBST and then incubated with fluorescent secondary antibodies (donkey anti-rabbit (780 nm), donkey anti-mouse (680 nm), 1:15000, Li-Cor Biosciences, Lincoln, NE, USA). Western blots were then imaged (Odyssey, Li-Cor Biosciences, Lincoln, NE, USA) and protein levels quantified with the Odyssey software. The hairpin plasmid with the highest knockdown efficacy (5′.AGTTCTTTGGTCTAGACTCCAATC.3′) was packaged into Adeno-Associated Virus2 (AAV2) by the University of North Carolina Gene Therapy Core Facility and used in subsequent behavioral tests. Control vector expressed a previously validated control hairpin not targeted to any gene (Hommel et al., 2003; Benzon et al., 2014).

### *In vivo* Knock Down of *Cyp26b1*

An AAV2-based vector that expresses *Cyp26b1* shRNA and eGFP or a non-targeted hairpin control vector (*n* = 10–11) was injected bilaterally into the rat NAc shell (1 μl/side over 10 min) using coordinates AP = 1.7, *L* = 2.2, *D* = -6.7. To validate the knockdown efficiency of AAV *in vivo*, AAV-control shRNA or AAV-*Cyp26b1* shRNA (*n* = 6) was injected in the nucleus accumbens shell (NAcSh) in rats. Two microliter of AAV was injected per side of the NAc to increase the number of infected neurons. NAc regions with eGFP fluorescence were collected and tissues from two rats were pooled together to increase the yield of protein concentration. The expression of CYP26B1 protein level was detected using western blot described above. For behavioral tests, an AAV2-based vector that expresses *Cyp26b1* shRNA and eGFP, or control vector (*n* = 10–11 each) was injected bilaterally with 1 μl/side into the rat NAc. Pair-housed rats were used instead of isolated rats for this study to increase relevance to the scientific community by demonstrating the effects of *Cyp26b1* independent of the EC/IC procedure. Behavioral tests started 5 weeks after stereotaxic surgery (**Figure 7**). Accurate placement was verified immunohistochemically after the conclusion of behavioral testing.

### Cocaine Self-Administration

#### Acquisition

One week after catheter surgery, all rats were placed in operant chambers (30 cm × 24 cm × 21 cm; Med-Associates, St. Albans, VT, USA) and allowed to self-administer 0.2 mg/kg/infusion unit dose of cocaine for 2 h per session for 5 days; then 0.5 mg/kg/infusion for 3 days on a fixed ratio (FR1) schedule. Each infusion was delivered intravenously in a volume of 0.1 ml over 5.8 s. The infusion was signaled by illumination of two cue lights for 20 s, which signaled a timeout period during which no further infusions could be attained. ***Fixed ratio dose response****:* Each rat was allowed to self-administer 0.5, 0.25, 0.125, 0.06, 0.03, 0.015, 0.0075, 0.00325 mg/kg/infusion cocaine in descending order on an FR1 schedule each day for five consecutive days. Rats self-administered each dose of cocaine for 30 min. ***Cue responding***: Rats were subjected to forced abstinence in their home cages for 7 days. On the 8th day, rats were placed in the operant chamber and allowed to self-administer **saline** under an FR1 schedule for 1 h with cue light presentation contingent on bar pressing. ***Extinction***: Stably responding rats underwent a within-session extinction procedure for 3 days. All rats were allowed to self-administer 0.5 mg/kg/infusion cocaine under an FR1 schedule for 1 h followed by extinction for 3 h. During the extinction period, lever responding resulted in cue-light illumination under an FR1 schedule, but the infusion pump did not deliver cocaine. ***Reinstatement***: All rats received 0.5 mg/kg/infusion unit dose of cocaine under an FR1 schedule for 1 h followed by 3 h of extinction. Next, all rats received an IP injection of cocaine of one of five doses (0, 2.5, 5, 10, 20 mg/kg) in a random order for each rat across the five sessions, followed by 3 h reinstatement responding session.

### Immunohistochemistry

For **Figure 7D**, the placement of AAV-*Cyp26b1*shRNA expression *in vivo* was validated by immunofluorescence staining with eGFP. The brains were extracted, post fixed, cryoprotected and sectioned into 40 μm slices containing the NAc on a sliding freezing microtome (Leica Biosystems, Richmond, IL, USA). The slices remained floating and were rinsed with 1xPBS prior to blocking with 3% normal donkey serum (Jackson ImmunoResearch, West Grove, PA, USA) with 0.3% triton. NAc slices were incubated with eGFP primary antibody overnight (1:500, chicken, Aves labs, Tigard, OR, USA) with 3% donkey serum, 0.3% triton in 1xPBS. After washing, slices were incubated with secondary Alexa 488 donkey anti chicken antibody (Jackson ImmunoResearch, West Grove, PA, USA) in 1xPBS. Finally, slices were mounted, dehydrated using ethanol and CitriSolv (Fischer Scientific, Waltham, MA, USA) and coverslipped with DPX (Fisher Scientific).

### Statistical Analysis for Behavior

Two-factor analyses of variance (ANOVAs) and two-factor repeated-measures ANOVAs were performed to compare four treatment groups. Significance between only two conditions was analyzed using a Student’s *t*-test. All *t*-test data passed the Shapiro–Wilk test of normality. All data are expressed as mean ± SEM. Statistical significance was set at *p* < 0.05.

## Results

### Descriptive Statistics

Raw and processed RNA sequencing data can be found in the Gene Expression Omnibus database with the project number GSE88736.

After the primary data alignment and analysis, 14,309 transcripts were quantified as the result of RNA-seq. The first step of our analysis was to investigate the regulation of individual transcripts. Based on the likelihood *F*-tests, the Venn diagram shows 106 transcripts significantly regulated (*p* < 0.001) by cocaine, 683 transcripts significantly regulated by environmental enrichment and 64 transcripts significant for the interaction (**Figure 1B**). In addition, the Venn diagram also displays the number of transcripts common among the effects, which indicates the overlapping effects of cocaine and environmental enrichment. Note that there are more transcripts upregulated than downregulated by cocaine, while there are many more downregulated transcripts than upregulated by environmental enrichment (**Figure 1C**).

In the significantly regulated gene lists, we first looked at the top 50 upregulated and downregulated transcripts from cocaine and environmental enrichment. These transcripts give the highest confidence of regulation. For cocaine (**Figure 1D**), immediate early genes such as EGR4, NR4A3, FOS, EGR2 were induced, which agrees with previous publications (Hope et al., 1992; Berke et al., 1998; Werme et al., 2000; Guez-Barber et al., 2011). For enrichment, the top regulated transcripts include transcription factors, such as NR4A1, EGR3, ARC, EGR2 and NR4A3, etc. (**Figure 1E**). In our search for a therapeutic target, we moved beyond individual transcripts to explore molecular pathways.

### Cocaine Effects on Transcription in the NAc

**FIGURE 2:**
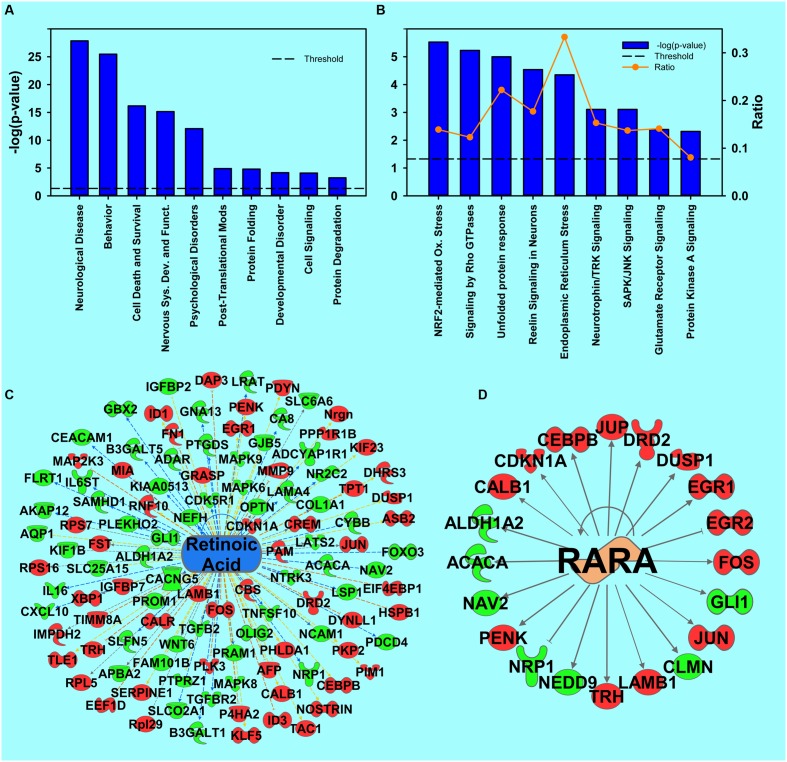
**Transcripts regulated by cocaine.** **(A)** Selection of significantly regulated Biological Functions and Diseases by cocaine as determined by IPA. The *y*-axis represents –log (*p*-value). Dotted line represents the threshold as *p*-value = 0.05. **(B)** A selection of significantly regulated Canonical Pathways by cocaine. Orange line represents a ratio of regulated transcripts to all transcripts in the pathway. **(C,D)** Transcripts from Upstream Regulator analysis whose expression predicts repressed signaling of retinoic acid **(C)** and RARα **(D)** by cocaine. Red represents upregulation; green represents downregulation.

To investigate which biological functions and cellular pathways are regulated by cocaine, significantly regulated transcripts were analyzed with IPA. **Figure 2A** lists some top-ranked diseases and biological functions of interest for the cocaine main effect. Complete IPA data can be found in the supplemental information.

#### Data Validation

In large data sets such as RNA sequencing, some orthogonal data cross-validation is important to provide confidence in the validity of the results. One approach of validating the RNA-seq data is to compare results with previous cocaine studies. In the top-ranked canonical pathways (**Figure 2B**), *Signaling by Rho GTPases* is represented in **Supplementary Figure S1** and was previously shown to be repressed by cocaine in the NAc (Kim et al., 2009; Gourley et al., 2011). The *Endoplasmic Reticulum Stress* pathway identified in the current results was confirmed previously (Pavlovsky et al., 2013). In the Upstream Regulator analysis, cocaine and *Creb1* are predicted to be upstream regulators by the IPA analysis (**Supplementary Figures S1B,C**). Full results of the upstream regulator analysis are presented in table form in the supplemental information. Additionally, *Depressive Disorder* and *Anxiety Disorders*, which show comorbidity with cocaine abuse (Sonne et al., 1994; Morton, 1999), were highlighted (**Supplementary Figure S1D**).

#### Retinoic Acid Pathway

One novel pathway highlighted in the cocaine main effect is RA signaling. Specifically, *Retinoic acid receptor (RAR) activation* was identified as a regulated canonical pathway (*p* = 0.023). Further, in the upstream regulator analysis, RA (*p* = 9.78E-10, activation *Z*-score = -1.553; **Figure 2C**), RA receptor α (RARA; *p* = 1.69E-7, *Z*-score = 0.723; **Figure 2D**), and the RARγ agonist CD437 (*p* = 4.25E-4, *Z*-score = -3.15, data not shown) were predicted to be upstream regulators of the cocaine main effect, all suggesting that RA signaling is important for the effects of cocaine. Full results of the upstream regulator analysis are presented in table form in the supplemental information. A secondary proteomic analysis from our previously-published report (Lichti et al., 2014) confirms CD437 as an upstream regulator at the protein level (*p* = 7.70E-4, *Z*-score = -2.23; **Figure 5**).

#### Other Functions and Pathways

In our results, one of the most regulated Diseases and Biological Functions from the IPA analysis is *Neurodegeneration-related Disorders*. In the Upstream Regulator analysis, 4 top predicted upstream regulators from the cocaine main effect are involved in neurodegeneration (**Supplementary Figure S2**), including amyloid precursor protein (APP; *p* = 2.66E-13), microtubule-associated protein tau (MAPT; *p* = 4.36E-7), presenilin 1 (PSEN1; *p* = 4.11E-7) and huntingtin (HTT; *p* = 1.16E-13). Additionally, the Canonical Pathway *Huntington’s Disease* signaling (*p* = 0.000282) is significantly regulated.

### Environmental Enrichment Effects on Transcription in the NAc

**FIGURE 3:**
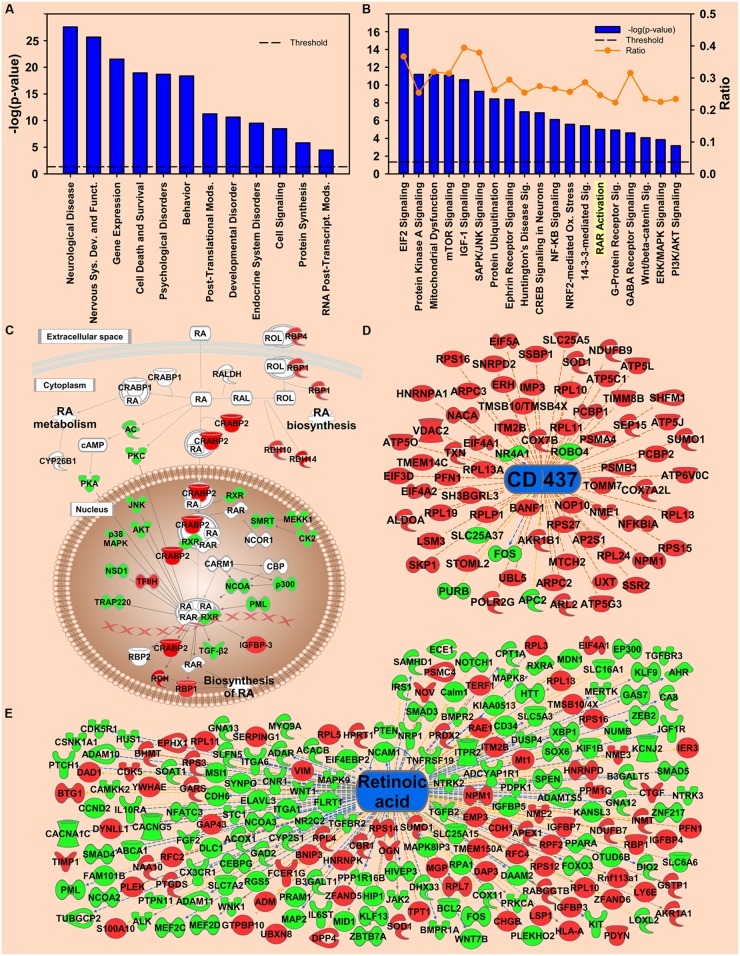
**Transcripts regulated by environmental enrichment.** **(A)** Selection of significantly regulated Biological Functions and Diseases by environmental enrichment as determined by IPA. The *y*-axis represents –log (*p*-value). Dotted line represents the threshold *p* = 0.05. **(B)** Selection of significantly regulated canonical pathways by environmental enrichment as determined by IPA. Orange line represents the ratio of regulated transcripts to all transcripts in the pathway. **(C)** Canonical pathway showing regulation of transcripts for retinoic acid signaling by environmental enrichment. **(D,E)** Transcripts from Upstream Regulator analysis whose expression predicts repressed signaling of CD437 **(D)** and retinoic acid **(E)** by environmental enrichment. Red represents upregulation; green represents downregulation.

To explore the molecular mechanism of environmental enrichment, IPA and GSEA were used to analyze the biological functions and pathways. For the environmental enrichment main effect, many mental disorder-related functions and diseases were significantly regulated (**Figure 3A**). The top-ranked canonical pathways involved *Protein Translation-Related EIF2 Signaling, PKA Signaling, Mitochondrial Dysfunction, Kinase Signaling*, etc. (**Figure 3B**). Complete IPA data can be found in the supplemental information.

#### Retinoic Acid Signaling Pathway

The RA signaling pathway was also significantly regulated by environmental enrichment (*p* = 9.77E-06) (**Figure 3B**). Within the RA signaling pathway, the transcripts involved in RA synthesis and translocation are upregulated by environmental enrichment, such as retinol binding proteins 1 and 4 (*Rbp1* and *Rbp4*), retinol dehydrogenase 10 (*Rdh10*), and cellular RA binding protein 2 (*Crabp2*), while the repressors of this pathway, such as the kinases *Akt* and *Pkc* are mainly downregulated (**Figure 3C**). Additionally, 215 RA target genes are regulated by environmental enrichment, most being downregulated (*p* = 1.84E-5, activation *Z*-score = -2.655; **Figure 3E**). Further, the agonist of the RA receptor γ, CD-437, was predicted to be inhibited as an upstream regulator of enrichment (*p* = 3.63E-18, activation *Z*-score = -7.541; **Figure 3D**). A secondary analysis of protein data from these rats (Lichti et al., 2014) confirms CD437 as an upstream regulator (*p* = 4.83E-15, *Z*-score = -3.13; **Figure 5**), highlighting the importance of RA signaling for further study.

#### Other Functions and Pathways

One of the most striking environmental enrichment effects was the regulation of transcription. The Gene Ontology gene set containing 217 transcription factor-related genes suggests a decrease in EC rats (**Supplementary Figure S3A**; NES = 1.67, *p* = 0.004). At the top of this gene set, the transcription of *Egr* transcription factors was highly repressed by environmental enrichment. In support of a functional effect on EGR transcription factors, *Egr1/2/3* target genes were also significantly repressed by environmental enrichment, as shown by a GSEA analysis of the Transcription Factor Target gene set V$EGR_Q6 (**Supplementary Figure S3B**; NES = 1.64, *p* < 0.001). Complete GSEA results can be found in the Supplementary Information. IPA also highlighted the roles of transcription factors. In the Upstream Regulator analysis, *Fos. Esrra* and *Srf* were identified as likely upstream regulators of the transcripts regulated by environmental enrichment. Differentially regulated *Fos* target genes revealed that *Fos* activity was enhanced in environmental enrichment despite the mRNA for *Fos* being down at 3 h (**Supplementary Figure S3C**; *p* = 1.06E-07, activation *z*-score = 1.420). In addition, *Esrra* (**Supplementary Figure S3D**; *p* = 2.11E-3, activation *z*-score = 2.668) was also identified as an activated upstream regulator. This result agrees with our prior research that *Esrra* was identified as an upstream regulator with energy metabolism proteins regulated in the proteomic study from tissue from these same rats (Lichti et al., 2014). Another transcription factor that was identified as upstream regulator was *Srf* (**Supplementary Figure S3E**; *p* = 2.34E-5, activation *z*-score = 0.101).

Beyond transcription, *EIF2 signaling* (*p* = 5.25E-17) was the top regulated canonical pathway (**Figure 3B**). Transcription of *Eif3. Eif4a*, and 39 different 40S and 60S ribosomal subunits were increased by environmental enrichment while transcription of the *Eif2* inhibitor, *Gsk3β*, and the phosphatase of *Eif2b* activator, *Erk*, were downregulated (**Supplementary Figure S4A**).

In addition to protein synthesis, mRNA for the *Protein Ubiquitination* pathway (*p* = 3.63E-09) was also significantly differentially regulated by environmental enrichment (**Supplementary Figure S4B**). These results were confirmed by the GSEA of the Gene Ontology gene set for *Proteasome Complex* (NES = 1.50, *p* = 0.024). In the protein ubiquitination process, target polyubiquitinated proteins undergo either degradation by the proteasome or de-ubiquitination by de-ubiquitinating enzymes (DUBs). Our results show an increase in transcription of ubiquitin C (UBC) and many proteasomal subunits with a coordinated decrease in transcription of DUBs (**Supplementary Figures S4B,C**), indicating that the enrichment condition likely enhances protein degradation through ubiquitination, an effect in agreement with our prior investigations of the proteomics of environmental enrichment (Fan et al., 2013b; Lichti et al., 2014). In addition to the pathway analysis, the role of UBC is highlighted in the Network analysis by IPA (**Supplementary Figure S4D**, network score = 35). Transcription of 21 out of 35 UBC target genes was upregulated by environmental enrichment. Sumoylation and ubiquitination have an important crosstalk in determining protein fate (Gill, 2004; Ulrich, 2012; Sriramachandran and Dohmen, 2014). SUMO proteins 1, 2, and 3 are the major hubs in another network determined by IPA (**Supplementary Figure S4D**), with 29 of 44 transcripts downregulated.

### Cocaine X Environment Interaction

**FIGURE 4:**
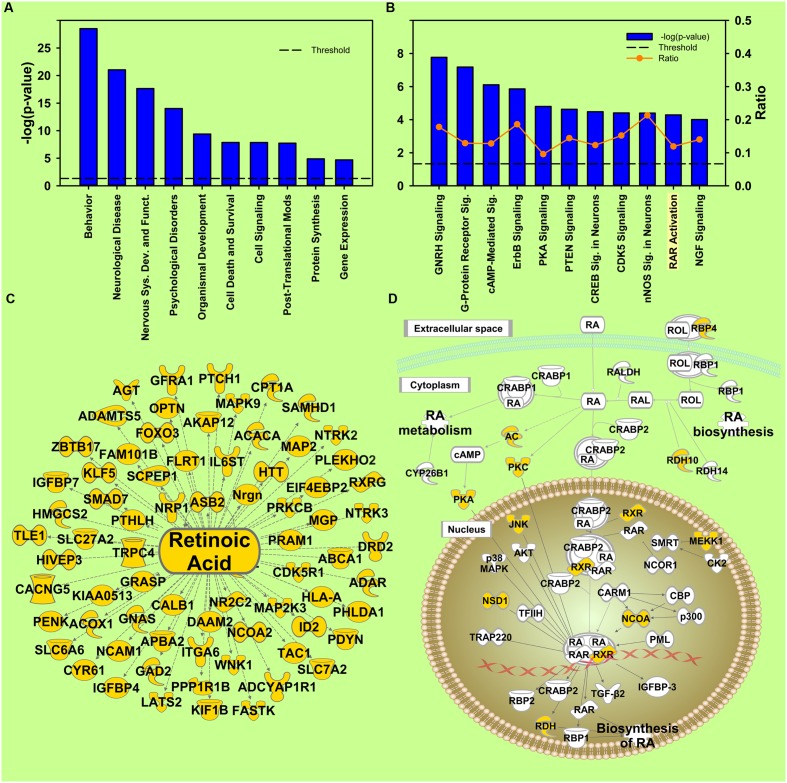
**Cocaine X Environmental Enrichment interaction.** **(A)** Selection of significantly regulated Biological Functions and Diseases. The *y*-axis represents –log (*p*-value). Dotted line represents the threshold *p* = 0.05. **(B)** Selection of significantly regulated canonical pathways. Orange line represents a ratio of regulated genes to all genes in the pathway. **(C)** Transcripts from Upstream Regulator analysis of the Environmental Enrichment X Cocaine interaction whose expression predicts regulation by retinoic acid. **(D)** Canonical pathway showing regulation of transcripts for retinoic acid signaling. Yellow represents differential regulation in EC and IC rats in response to cocaine.

The transcription regulated by the interaction indicates that EC and IC rats respond differently to cocaine (**Figure 4**). Studying this differential response helps to identify the molecular mechanisms of the protective EC phenotype. For the interaction, *Drug Dependence* (*p* = 3.38E-6; **Supplementary Figure S5A**) and *Release of Dopamine* (*p* = 5.41E-7; **Supplementary Figure S5B**) were top-regulated diseases and biological functions in IPA. Regulated transcripts in *Drug Dependence* were dominated by ion channels and G-protein coupled receptors (GPCRs). *Release of Dopamine* was also dominated by GPCRs.

**FIGURE 5:**
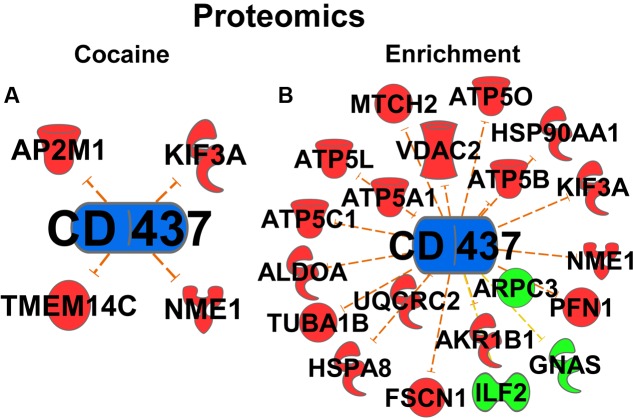
**Proteomic Upstream Regulator analyses.** Cocaine **(A)** and Environmental Enrichment **(B)** induce protein expression of target genes of CD437 (an Rarγ agonist).

#### Retinoic Acid Signaling Pathway

Retinoic acid receptor (RAR) activation was also identified in the Canonical Pathway analysis (*p* = 5.13E-05) in the interaction of Cocaine and Enrichment (**Figure 4B**). Some essential genes in this pathway showed significant interaction at the mRNA level, such as retinol binding protein (*Rbp4*), retinol dehydrogenase (*Rdh10*), retinoid X receptor (*Rxr*), etc. (**Figure 4D**). Additionally, RA was identified as an upstream regulator (*p* = 1.6E-2; **Figure 4C**). These results indicate that the activity of the RA signaling pathway and the transcripts of RA target genes are differentially regulated by cocaine in EC and IC rats, and are therefore a promising avenue for developing novel addiction therapeutics.

#### Other Functions and Pathways

Protein kinases play a significant role in post-translational modification to activate or inhibit target proteins through phosphorylation. For the interaction, *Activation of Protein Kinases* was also identified as differently regulated (*p* = 1.81E-6; **Supplementary Figure S5C**). This prediction is based on the regulation of kinase and kinase related transcripts, including 6 different mitogen-activated protein kinases (MAPKs; **Supplementary Figure S5D**). In addition to the expression of kinases in general, *Protein Kinase A Signaling* (*p* = 1.6E-05) ranked 12th among the regulated canonical pathways. Our results also show that corticosterone was identified as an upstream regulator in the NAc (*p* = 8.66E-4; **Supplementary Figure S5F**). This result is not surprising because it has been found that EC rats have blunted induction of corticosterone induced by psychostimulants (Stairs et al., 2011; Crofton et al., 2015). One important function in the NAc that responded differently to cocaine in EC and IC rats was *Transport of Ca^2+^* (*p* = 3.67E-5; **Supplementary Figure S5G**). Angiotensinogen (AGT), ATPase (ATP2B4) and voltage-dependent calcium channel (CACNA1G) lead to activation of transport of Ca^2+^, while parathyroid hormone-like hormone (PTHLH) and arginine vasopressin (AVP) lead to inhibition (**Supplementary Figure S5G**). In addition to the above functions, *NMDA receptor* downstream transcripts (*p* = 2.72E-6; **Supplementary Figure S5E**) also responded differently to cocaine in EC and IC rats.

### Validation of Quantitative RNA Sequencing

To confirm the validity of the quantitative RNA sequencing technique, real-time PCR was used to quantify the mRNA expression of *Fabp5* and *Hspa5* from the same rats. mRNA fold change results from RNA sequencing and qPCR of *Fabp5* (*R*^2^ = 0.5155, *p* < 0.0001) and *Hspa5* (*R*^2^ = 0.4301, *p* = 0.0003) was compared for every rat. These results indicate qPCR and RNA sequencing results are well correlated in addition to the orthogonal validation of comparing the current results against previous cocaine and enrichment findings.

### Regional Enhancement (NAc shell) of Retinoic Acid-Related Genes

**FIGURE 6:**
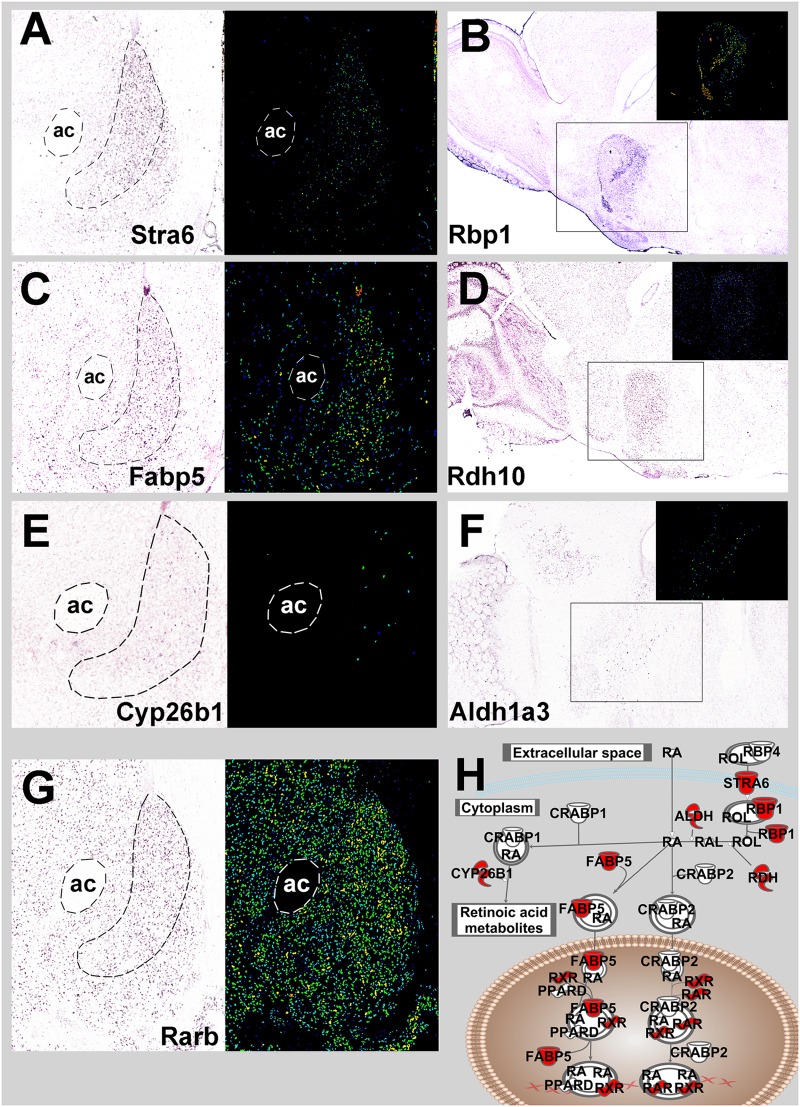
**Mouse NAc shell-restricted expression of genes involved in retinoic acid signaling pathway from Allen Brain Atlas.** **(A)** Coronal section of *Stra6* NAc *in situ* mRNA expression. Bright field image is on the left and the identical signal image is to the right. ac, anterior commissure. **(B)** Sagittal section of *Rbp1 in situ* mRNA expression. Signal image is inset. **(C)** Coronal section of *Fabp5* expression. **(D)** Sagittal section of *Rdh10* expression. **(E)** Coronal section of *Cyp26b1* expression. **(F)** Sagittal section of *Aldh1a3*. **(G)** Coronal section of *Rarβ* expression. (**A–G** Images are from Allen Brain Atlas) **(H)**. Red symbols denote retinoic acid-related genes with NAc shell-specific expression.

A topographic transcriptomic analysis for genes with selectively enhanced gene expression in the NAc shell from the Allen Brain Atlas identified 178 transcripts with selective expression in the NAc shell ≥ 1.25 fold over surrounding regions. An IPA analysis of these 178 genes revealed the central RA pathway as being significant with Stimulated by RA 6 (*Stra6)*, Retinoic acid receptor β (*Rarb*), Fatty Acid Binding Protein 5 (*Fabp5*) and Cytochrome P450, Family 26, Subfamily B, Polypeptide 1 (*Cyp26b1*) as shell-selective genes (*p* = 1.67E-5; **Figure 6**). A manual search of NAc shell-selective RA gene expression in sagittal sections identified *Rbp1. Rdh10* and *Aldh1a3* as additional selective genes. A search of the literature confirmed *Rbp1* and *Rarb* selective expression, along with *Aldh1a1* and *Rxrg* (McCaffery and Drager, 1994; Zetterstrom, 1999). These nine NAc shell-enhanced genes are depicted in the pathway for **Figure 6H**.

### RA Signaling in NAc Shell Increases Cocaine Self-Administration

**FIGURE 7:**
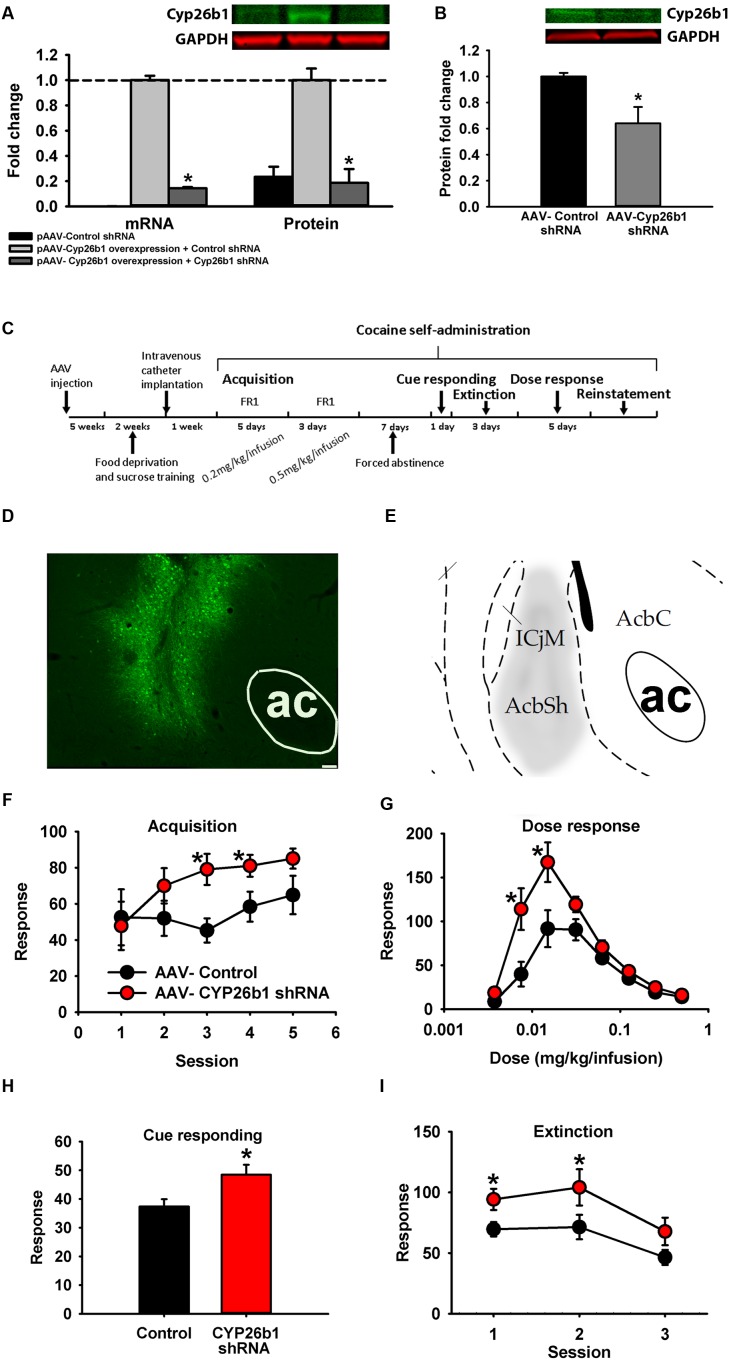
**Knocking down *Cyp26b1* in rat nucleus accumbens increases cocaine self-administration.** **(A)** Fold change of Cyp26b1 mRNA (±SEM) and protein (±SEM) in HEK293 cells transfected with pAAV-non-targeted control shRNA, pAAV- non-targeted control shRNA and Cyp26b1 overexpression plasmid, or pAAV-Cyp26b1shRNA and Cyp26b1 overexpression plasmid. **(B)** Fold change of Cyp26b1 protein (±SEM) in rat NAc shell injected with AAV- non-targeted control shRNA or AAV-Cyp26b1shRNA. **(C)** Schematic diagram of experimental procedure for vector behavioral tests. **(D)** Representative *in vivo* titer from AAV-*Cyp26b1* shRNA vector co-expressing eGFP. Bar represents 75 μm. **(E)** Stereotaxic atlas (Paxinos and Watson, 2005) defining region targeted. **(F–I)** Knocking down *Cyp26b1* in NAc increases response rate for acquisition of cocaine self-administration at 0.2 mg/kg/infusion in 2-h sessions **(F)**, maintenance response rate at low unit doses (30min/dose) **(G)**, cue response rate (1 h) **(H)** and increases in extinction responding (1-h FR1 session followed by 3-h extinction) **(I)**. (^∗^*p* < 0.05; *n* = 9–10/group).

Two strategies for changing concentrations of RA are to either alter synthesis or degradation. However, because there are several different subtypes of *Rdhs* and *Raldhs*, knocking down any one could be compensated by the other RA synthases. Therefore, we decided to alter RA concentration by knocking down the degradation enzyme, *Cyp26b1*, since RA signaling was regulated by cocaine, enrichment, and their interaction, and many genes showed selectively enhanced expression in the NAc shell. With decreased expression of *Cyp26b1*, RA subsequently builds up in neurons, enhancing RA downstream signaling (Kim et al., 2014). To knock down the expression of *Cyp26b1*, an shRNA targeting the *Cyp26b1* coding sequence was designed and knockdown efficiency was examined *in vitro* and *in vivo*. Compared with a non-targeted control shRNA, *Cyp26b1* shRNA significantly decreased expression of *Cyp26b1* in HEK293 cells in both mRNA and protein (**Figure 7A**) and in rat NAc shell at the protein level (**Figure 7B**). **Figure 7C** shows the schematic diagram of the experimental timeline for behavioral tests. For behavioral testing, AAV-*Cyp26b1*shRNA or control vector was injected into the NAc shell of rats (**Figure 7D**, atlas comparison **Figure 7E**). For acquisition of cocaine self-administration, results demonstrate significant acquisition across sessions [*F*(4,80) = 3.855, *p* = 0.006; **Figure 7F**] with a trend for increased acquisition in *Cyp26b1* shRNA rats [interaction *F*(4,80) = 2.142, *p* = 0.083]. A *t*-test showed that *Cyp26b1* shRNA rats responded significantly more for Sessions 3 and 4. For maintenance responding (**Figure 7G**), knocking down *Cyp26b1* significantly increased responding for cocaine at low unit doses, resulting in significant main effects of Dose [*F*(7,26) = 39.239, *p* < 0.001], Vector [*F*(1,18) = 7.938, *p* = 0.011] and a significant interaction [*F*(7,126) = 4.871, *p* < 0.001]. Knockdown significantly elevated the responding for cues in a cue responding test [**Figure 7H**; *t*(20) = -2.572, *p* = 0.018]. Finally, in a within-session extinction procedure (**Figure 7I**), rats with *Cyp26b1* knockdown exhibited increased responding compared to control rats, with significant main effects of Session [*F*(2,36) = 15.317, *p* < 0.001] and the Vector main effect is at the threshold of the *p*-value cutoff for significance [*F*(1,18) = 4.433, *p* = 0.05], indicating that knockdown of *Cyp26b1* in the NAc shell enhances drug-seeking behavior. In cocaine-induced reinstatement, high variance prevented detection of a difference in reinstatement between the two groups of rats at any dose.

## Discussion

These studies highlight mechanisms of the protective addiction phenotype of environmental enrichment and identify novel targets that play a role in regulating addiction-related behavior. Among the novel molecules and pathways identified, the RA signaling pathway was predicted to play an important role in the differential response to cocaine in EC and IC rats. Separately, a topographic transcriptomic analysis identified RA-related genes and RA target genes as being selectively expressed in the NAc shell, further highlighting the likely importance of RA in addiction-related behavior. These results generated a hypothesis-driven experiment that confirmed the role of RA in addiction-related behavior.

### Cocaine Transcriptomic Effects

In the upstream analysis of cocaine-regulated transcripts, cocaine itself and CREB1, an important mediator of the effects of psychostimulants (Carlezon et al., 1998; Pliakas et al., 2001), ranked at the top of the list as upstream regulators, strongly supporting that cocaine-regulated transcription seen here agrees with previous studies. Our prior research demonstrated that enriched rats have less phospho-CREB in the NAc and that decreasing CREB function in the accumbens shell produces a behavioral phenotype identical to that of environmental enrichment (Bowling and Bardo, 1994; Green et al., 2002, 2010), an interesting behavioral phenotype marked by increased sensitivity to the rewarding effects of stimulants (as measured by CPP) coupled with decreased self-administration (Pliakas et al., 2001; Larson et al., 2011).

### Environmental Enrichment and Transcription

Compared to the cocaine main effect, there were approximately 5X more transcripts significantly regulated by environmental enrichment, revealing that environment has a much more extensive impact on gene expression than cocaine exposure. Transcription factors were the most regulated gene sets by environmental enrichment. Another impressive difference between EC and IC rats is the regulation of transcripts involved with EIF2 signaling. Even though *Eif2* itself is not regulated at the mRNA level, decreased upstream inhibitors of *Eif2* and upregulated downstream ribosomal subunits suggests regulation of the protein translation process. In addition to protein synthesis, the protein degradation system is also altered by enrichment. Prior research from this laboratory demonstrated that expression of ubiquitin target proteins is different in EC and IC rats (Fan et al., 2013a,b). Ubiquitination is also important in differential expression of proteins from the current rats (Lichti et al., 2014). The current mRNA data revealed increased transcription of ubiquitin and proteasomal subunits, but reduced mRNA expression for deubiquitinating enzymes in the NAc, possibly suggesting enhanced protein degradation in EC rats. Taken together, enhanced protein translation and degradation likely indicate more rapid protein turnover in EC rats compared with IC rats.

### Retinoic Acid Signaling

Retinoic acid genes are selectively expressed in the NAc shell, as shown by a topographic transcriptomic analysis of the Allen Brain Atlas and the published literature (McCaffery and Drager, 1994; Zetterstrom, 1999). Given the importance of the NAc shell to addiction, the RA signaling pathway offers promising targets for novel therapeutic development for cocaine addiction. One previous report found that constitutive whole-body RARβ/RXRβ or RARβ/RXRγ double null mice had a selective decrease of dopaminergic D2 receptors in the shell of the NAc, with concomitant decrements in locomotor and rotarod performance (Krezel et al., 1998). Thus far, however, there have not been any other systematic studies aimed at understanding RA signaling and addiction.

The current report provides converging evidence from the Upstream Regulator analysis (predicting function) of environmental enrichment and the vector knockdown of Cyp26b1 suggesting that RA signaling activity in the NAc shell increases susceptibility to drug taking. Given that every core component of the RA signaling pathway involves protein interactions with small molecules (i.e., retinoids) this pathway is a prime candidate for the development of selective small molecule inhibitors as possible pharmacotherapeutics for cocaine addiction. One advantage of choosing targets in this pathway is that nine components of this pathway have some enhancement of expression in the NAc shell (**Figure 6**), providing some degree of regional selectivity and thereby decreasing the likelihood of unwanted side effects. These regionally-enhanced components include the binding proteins *Stra6. Rbp1*, and *Fabp5*, the synthesis enzymes *Adh10. Aldh1a1*, and *Aldh1a3*, the degradation enzyme *Cyp26b1*, and the RA receptors *Rarβ* and *Rxrγ*. Ongoing experiments are investigating which of these targets would be most suitable for pharmacotherapeutic development.

## Conclusion

Environmental factors play a significant role in individual differences in responses to drugs of abuse. Although some transcription factors, such as ΔFosB and CREB, have been reported to mediate the protective addiction phenotype of environmental enrichment (Green et al., 2010; Zhang et al., 2014), RNA sequencing technology has produced a broader view of transcriptomic responses of the NAc in EC and IC rats after cocaine. Taken together, the discovery-based transcriptomic analyses and hypothesis-driven behavioral tests have revealed RA signaling as a novel mechanism involved in regulating the responses to both cocaine and environmental enrichment, revealing a novel pharmacotherapeutic target for the effective treatment of drug addictions.

## Author Contributions

TG, YZ, EC, and FK participated in the design of the work. TG, YZ, EC, FK, MS, DL, and XF participated in the acquisition, analysis, or interpretation of data for the work. All authors participated in the final approval of the manuscript and the review.

## Conflict of Interest Statement

The authors declare that the research was conducted in the absence of any commercial or financial relationships that could be construed as a potential conflict of interest.

## Footnotes

1 http://www.brain-map.org

2 http://www.ncbi.nlm.nih.gov/geo/query/acc.cgi?acc=GSE88736

3 http://mouse.brain-map.org/agea
