# Supplementary material for: Transcriptomics of Environmental Enrichment Reveals a Role for Retinoic Acid Signaling in Addiction
Source: Front Mol Neurosci. 2016 Nov 16;9:119. doi: 10.3389/fnmol.2016.00119 (PMC5110542; doi:10.3389/fnmol.2016.00119)
Supplement: Supplementary file 4 [file Image_1.PDF]

Supplemental Figures and Table

Figure S1

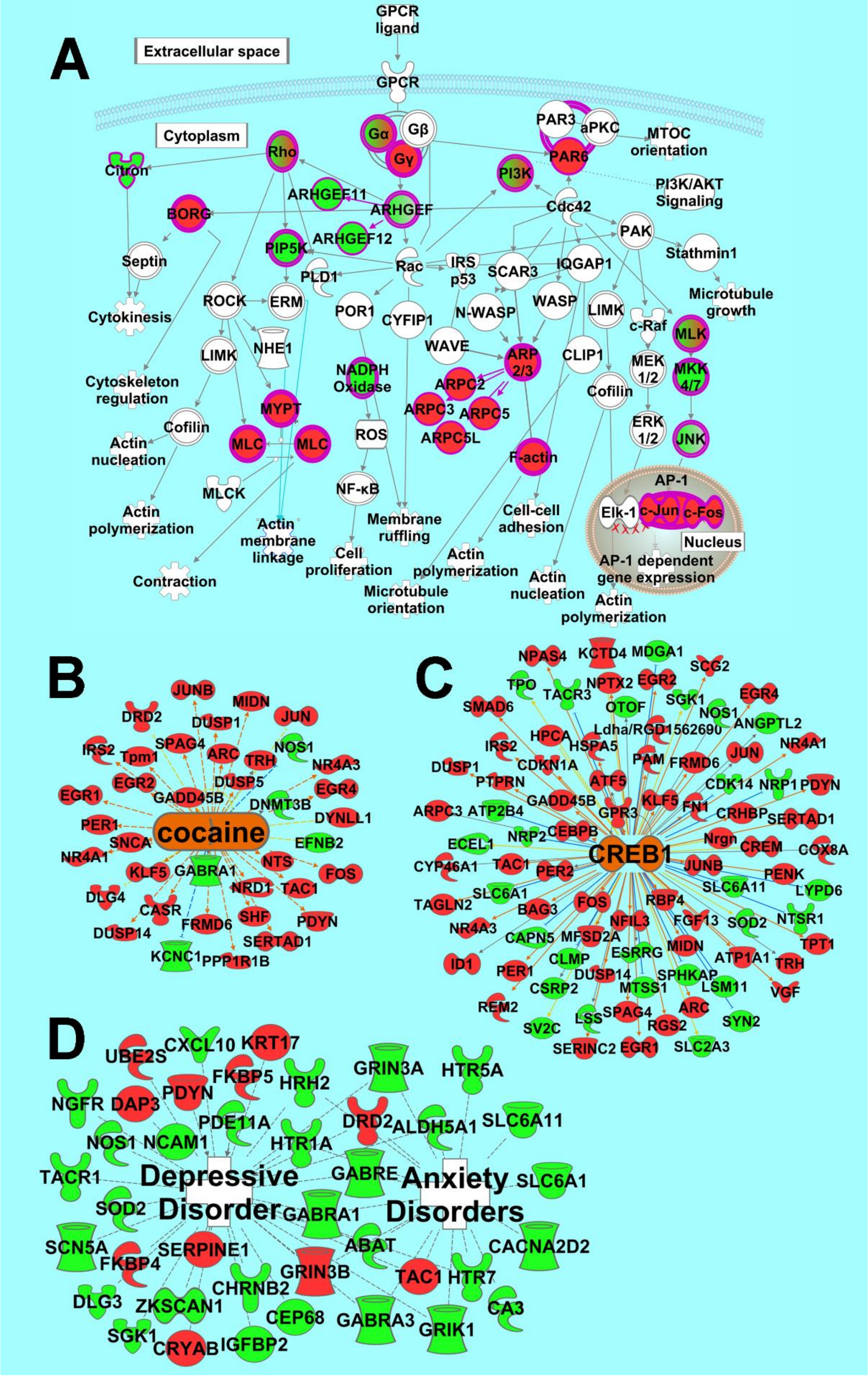

Supplementary Figure 1. Cocaine main effect. A. Signaling by Rho GTPases is regulated by cocaine in rat NAc determined by IPA. B. Validation of RNA-seq bioinformatics analysis confirming cocaine as an upstream regulator for cocaine-regulated transcripts. C. Transcripts from Upstream Regulator analysis in IPA with expression consistent with increased CREB activity. D. Cocaine-regulated transcripts associated with depression and anxiety disorders. Red represents upregulation by cocaine and green represents downregulation.

Figure S2

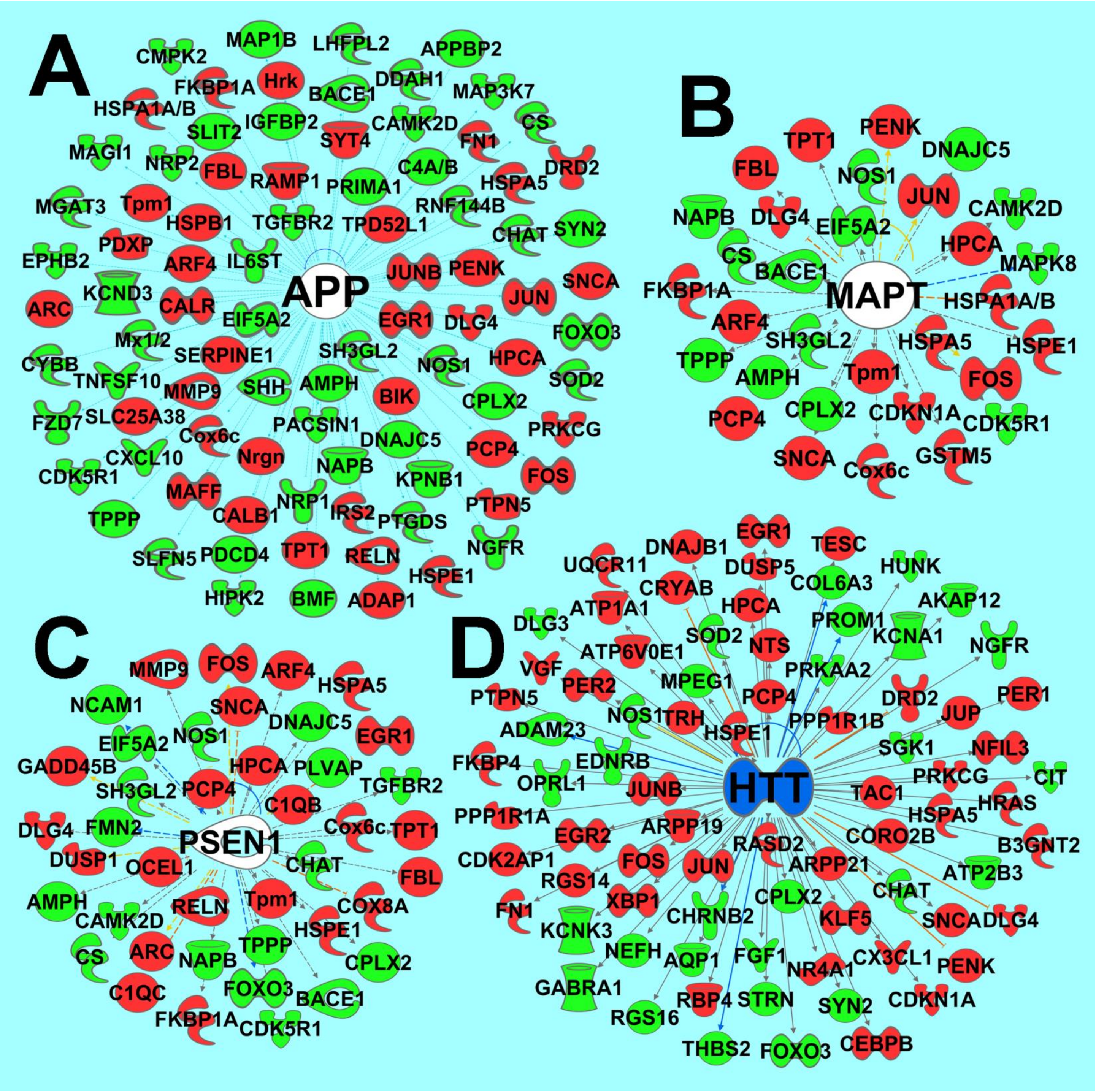

Supplementary Figure 2. Effects of cocaine on neurodegeneration gene expression. Transcripts from Upstream Regulator analysis in IPA showing expression of transcripts downstream of APP (A), MAPT (B), PSEN1 (C) and HTT (D). Red represents upregulation by cocaine and green represents downregulation.

Figure S3

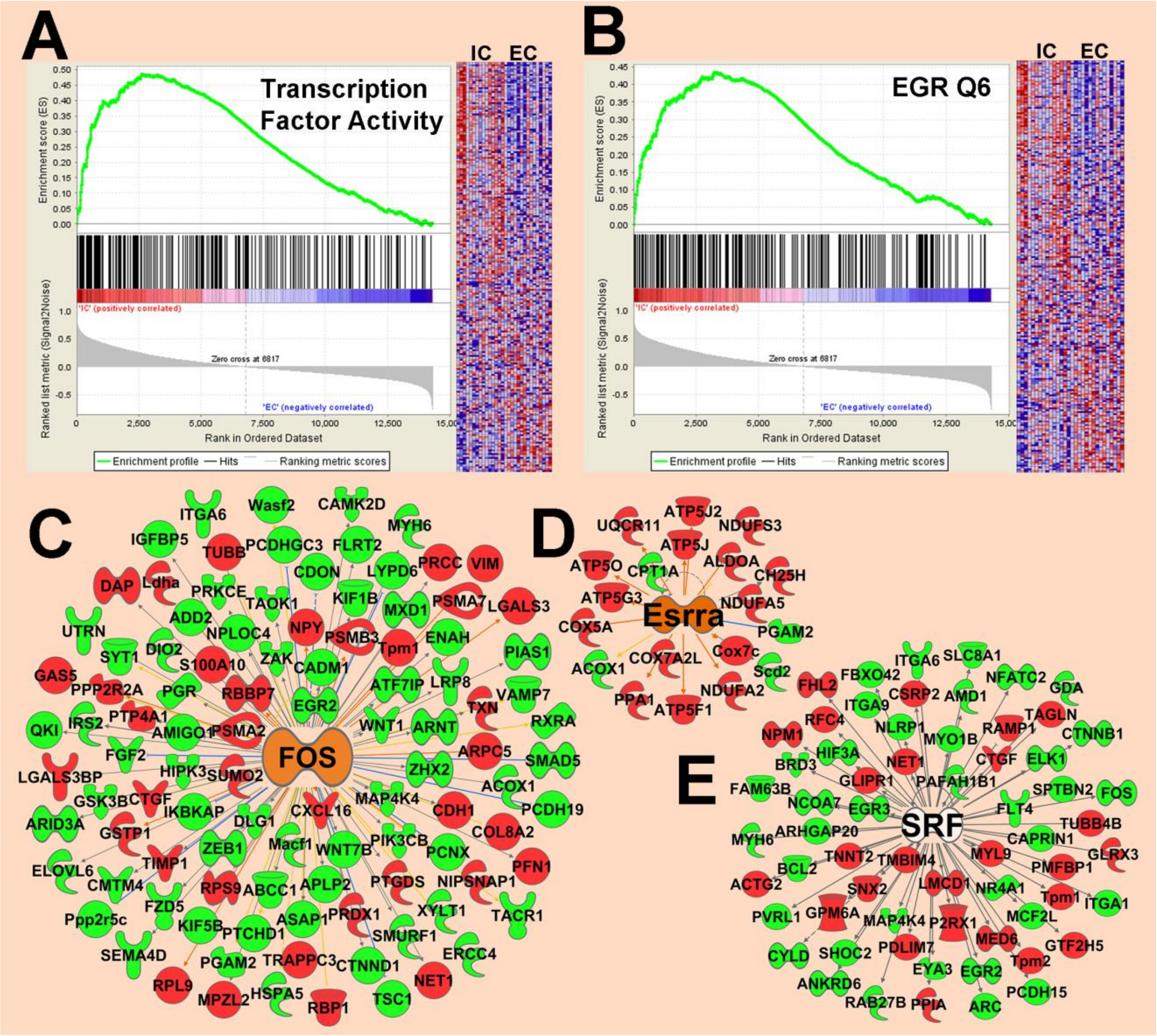

Supplementary Figure 3. Effects of environmental enrichment on transcripts for transcription factors. A-B. GSEA analysis showing decreased transcription of genes relating to the GO group for *Transcription Factor Activity* (A) and for conserved EGR target genes (B) in EC rats. Vertical ticks denote genes in the ranked list of genes. In the heat map, red represents high expression level and blue represents low expression level. C-E. Transcripts from the IPA Upstream Analysis whose expression supports regulation of cFos (C), Esrra (D), and SRF (E). Red symbols represent upregulation by environmental enrichment and green represents downregulation.

Figure S4

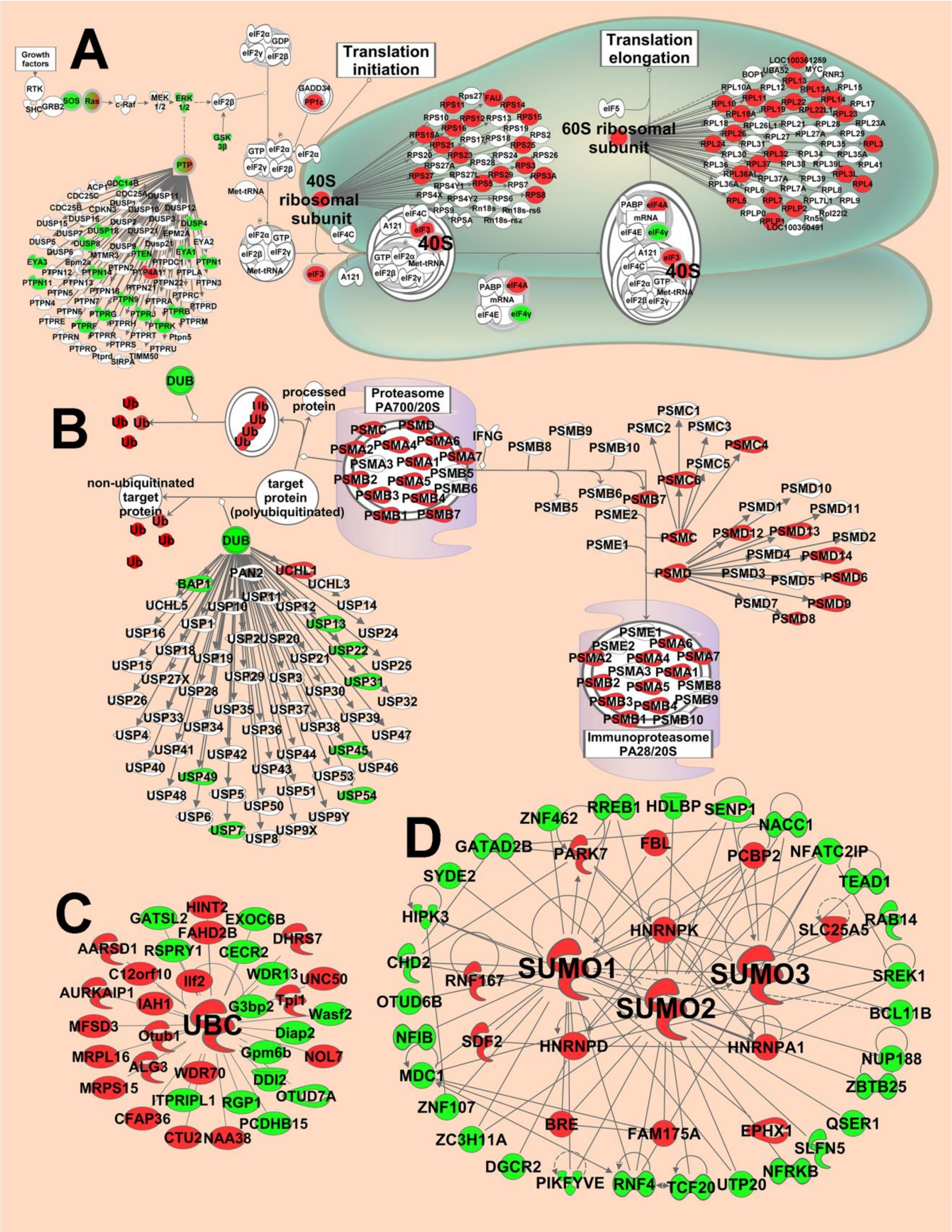

A
Growth factors
RTK
SHC
GRB2
SOS
Ras
c-Raf
MEK 1/2
ERK 1/2
eIF2β
GSK 3β
PTP
Translation initiation
GADD34
PP1c
Translation elongation
eIF5
40S ribosomal subunit
60S ribosomal subunit
40S
mRNA
PABP
eIF4A
eIF4E
eIF4γ
eIF4C
eIF3
A121
Met-tRNA
GTP
eIF2α
eIF2γ
GDP
B
DUB
Ub
processed protein
Proteasome PA700/20S
IFNG
non-ubiquitinated target protein
target protein (polyubiquitinated)
Immunoproteasome PA28/20S
PSMC
PSMD
PSME1
PSME2
C
UBC
D
SUMO1
SUMO2
SUMO3
HNRNPK
HNRNPD
HNRNPA1

Supplementary Figure 4. Effects of environmental enrichment on transcripts for protein translation and degradation. A. Canonical pathway for EIF2 signaling from IPA. Transcripts whose expression was increased in EC rats are highlighted in red, and those decreased in EC rats are highlighted in green. B. Canonical pathways for protein ubiquitination. C. Network of regulated transcripts with UBC and ubiquitin target genes. Red symbols denote upregulation in EC rats and green represents downregulation. D. Network of regulated transcripts identified SUMO1, 2 and 3 and changes in transcription of SUMO target genes.

Figure S5

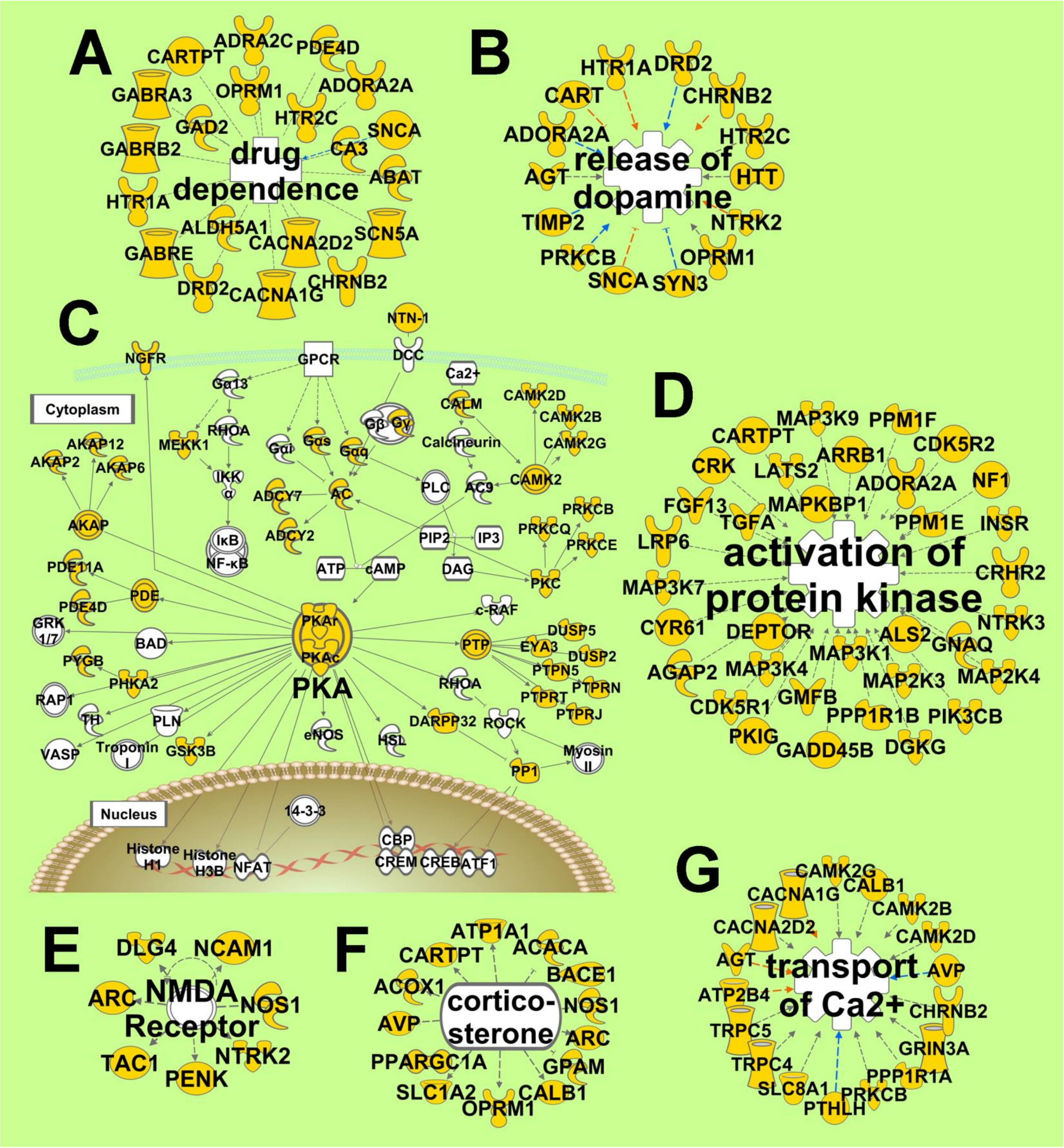

Supplementary Figure 5. Cocaine X Environmental Enrichment interaction. A. Transcription of genes involved in *Drug Dependence*. B. Transcription of genes involved in *Release of Dopamine*. C. Canonical pathway showing differential regulation of transcripts involved in the *PKA Signaling* pathway. Yellow symbols

are transcripts showing a significant cocaine x enrichment interation. D. Transcription of genes involved in *Activation of Protein Kinase*, a subset from the IPA Biological Functions analysis of *Post-translational modification*. E-F. Upstream Regulator analysis highlighting *NMDA receptor* (C) and *Corticosterone* (D) as regulators of transcript levels. G. Regulation of genes involved in *Transport of Calcium*, a subset from the IPA Biological Function analysis of *Molecular transport*.

Figure S6

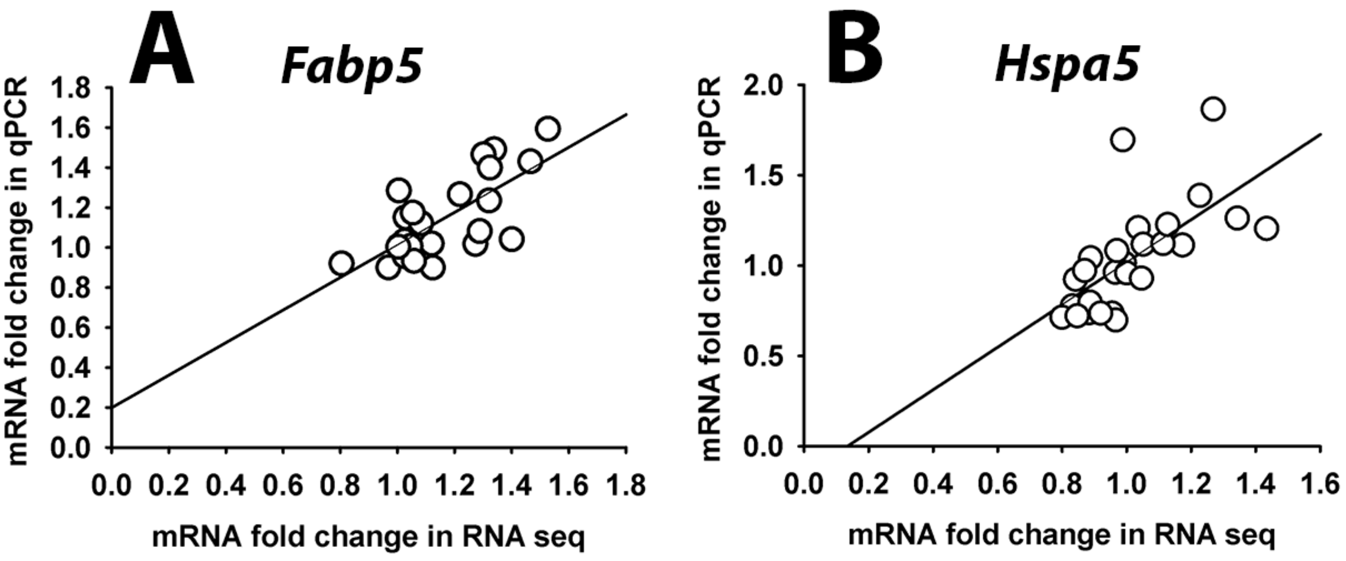

Supplementary Figure 6. Correlation between RNA sequencing and qPCR results. Correlation analysis of *Fabp5* (A) and *Hspa5* (B) mRNA fold change results from real-time PCR and RNA sequencing.

Table S1: shRNA targeting CYP26b1 coding sequence

Name	shRNA Sequence (24bp)	GC%	Start site
1	GCACATCCTTGATCATGCAACTAC	45.8	914
2	CGAACTGGATGGTTTCCAGATCCC	54.2	1137
3	AGTTCTTTGGTCTAGACTCCAATC	41.7	1466
4	AGACCTTCGAACTGGATGGTTTCC	50	1130
5	CCAGCACATCCTTGATCATGCAAC	50	911
